# Machine learning methods reveal the temporal pattern of dengue incidence using meteorological factors in metropolitan Manila, Philippines

**DOI:** 10.1186/s12879-018-3066-0

**Published:** 2018-04-17

**Authors:** Thaddeus M. Carvajal, Katherine M. Viacrusis, Lara Fides T. Hernandez, Howell T. Ho, Divina M. Amalin, Kozo Watanabe

**Affiliations:** 1 Department of Civil and Environmental Engineering, Ehime University, Matsuyama, Japan; 2 Biology Department, De La Salle University, Taft Ave, Manila, Philippines; 3 Biological Control Research Unit, Center for Natural Science and Environmental Research, De La Salle University, Taft Ave, Manila, Philippines; 4 Graduate School, University of the East Ramon Magsaysay Memorial Medical Center, Quezon City, Philippines

**Keywords:** Dengue, Modeling techniques, Weather, Relative humidity, Climate, Metropolitan Manila

## Abstract

**Background:**

Several studies have applied ecological factors such as meteorological variables to develop models and accurately predict the temporal pattern of dengue incidence or occurrence. With the vast amount of studies that investigated this premise, the modeling approaches differ from each study and only use a single statistical technique. It raises the question of whether which technique would be robust and reliable. Hence, our study aims to compare the predictive accuracy of the temporal pattern of Dengue incidence in Metropolitan Manila as influenced by meteorological factors from four modeling techniques, (a) General Additive Modeling, (b) Seasonal Autoregressive Integrated Moving Average with exogenous variables (c) Random Forest and (d) Gradient Boosting.

**Methods:**

Dengue incidence and meteorological data (flood, precipitation, temperature, southern oscillation index, relative humidity, wind speed and direction) of Metropolitan Manila from January 1, 2009 – December 31, 2013 were obtained from respective government agencies. Two types of datasets were used in the analysis; observed meteorological factors (MF) and its corresponding delayed or lagged effect (LG). After which, these datasets were subjected to the four modeling techniques. The predictive accuracy and variable importance of each modeling technique were calculated and evaluated.

**Results:**

Among the statistical modeling techniques, Random Forest showed the best predictive accuracy. Moreover, the delayed or lag effects of the meteorological variables was shown to be the best dataset to use for such purpose. Thus, the model of Random Forest with delayed meteorological effects (RF-LG) was deemed the best among all assessed models. Relative humidity was shown to be the top-most important meteorological factor in the best model.

**Conclusion:**

The study exhibited that there are indeed different predictive outcomes generated from each statistical modeling technique and it further revealed that the Random forest model with delayed meteorological effects to be the best in predicting the temporal pattern of Dengue incidence in Metropolitan Manila. It is also noteworthy that the study also identified relative humidity as an important meteorological factor along with rainfall and temperature that can influence this temporal pattern.

**Electronic supplementary material:**

The online version of this article (10.1186/s12879-018-3066-0) contains supplementary material, which is available to authorized users.

## Background

Dengue fever is a mosquito-borne disease transmitted by the bite of a female *Aedes aegyti* and *Ae. albopictus* mosquitoes, the primary and secondary mosquito vectors respectively, leading to clinical manifestations of influenza-like symptoms to life-threatening shock syndrome [1]. It had been estimated that 3.9 billion people in 128 countries or 40% of the world population are at risk of contracting the disease [2]. The dynamics of dengue transmission is said to be influenced by three central risk factors that are biological, sociological and environmental in nature [3]. The focus of our study is primarily on the environmental factors such as meteorological patterns that have been attributed to the spread and occurrence of dengue. A list of growing evidence has demonstrated the association between these factors and its epidemiology [4] thus claiming that dengue transmission is sensitive to meteorological variability and change [5–8]. Moreover, many researchers are asserting the possible future spatial and temporal expansion on the ecology of *Ae. aegypti* along with its virus in the midst of climate change scenarios [8–11].

Meteorological factors such as temperature, rainfall and humidity can influence dengue epidemiology by increasing mosquito development, population growth, virus replication and mosquito-human interactions [7]. Rainfall and flood have been shown to be important factors in providing appropriate breeding sites of *Ae. aegypti* necessary for increasing female mosquito density that may lead to outbreaks [12]. Temperature, on the other hand, has an effect in the reproductive and biting rate of the mosquito vector and the extrinsic incubation of the dengue virus [13]. Humidity tends to influence the vector’s longevity and rapid replication of the virus [14, 15]. In contrast to the three meteorological factors, wind speed or velocity has been shown to suppress mosquito flight, thus, affecting their oviposition and density [16–19]. Wind direction, on the other hand, had been explored [18] but there is no mention of how this meteorological variable may influence dengue transmission.

Analyzing the impact of meteorological factors to local dengue disease occurrence has become significant not only because it allows us to understand the role of these factors towards the spread of the disease, but also provides an opportunity to create and adopt an early warning dengue outbreak system [19, 20]. Many Southeast Asian countries had studied the role and association of meteorological variables towards its local dengue epidemiology. High dengue epidemics were considered seasonal and coincides the rainy season in Malaysia [21–23], Thailand [24, 25],Vietnam [26, 27] and the Philippines [28]. On the other hand, Singapore has shown that mean temperature, relative humidity [6, 29, 30] and Southern Oscillation index [31] were important meteorological factors in forecasting or predicting dengue outbreaks. Parts of Indonesia show that both temperature and rainfall are highly associated with high dengue incidence that can lead to epidemics [32]. In the Philippines, changing rainfall patterns [28], temperature and incidence of La Nina contribute varying dengue incidence [33].

The compendium of literature that assessed different meteorological effects on *Ae aegypti* and dengue incidence featured different statistical modeling techniques and used different dependent variables (e.g. current or lagged meteorological variables). The most commonly used modeling techniques are cross-correlations, Poisson Regression, Generalized Additive Modeling (GAM), Autoregressive Integrated Moving Average (ARIMA) [4] and distributed lag non-linear model (DLNM) [19, 34]. Most studies have used GAM and ARIMA models because it became the standard reference for associating environmental factors towards disease outcome and a tool for time series prediction analysis [35–37]. Although these statistical modeling techniques are widely used, they suffer certain drawbacks or disadvantages such as handling of missing values, outlier sensitivity, and multicollinearity [38]. More recently, new modeling approaches such as Machine learning methods are gaining popularity and interest because of its flexibility in handling complex and multiple interacting elements [39]. Our study focuses on two popular Machine Learning methods, Random Forest (RF) and Gradient Boosting (GB), because of its wide use in ecology and public health [40–42]. These two approaches are designated as tree-based algorithms because of its fundamental process in splitting the dataset into two or more homogeneous sets based on the most significant input variables. The end-result is a tree-like diagram where each node has a partitioning rule [43]. Tree-based learning algorithms are considered to be the best and widely used machine learning methods in generating a model of high and accurate prediction [44, 45]. Previous studies which used these modeling approaches (RF and GB) were able to predict the occurrence of dengue using either clinical (e.g. complete blood counts, symptoms) [46–48] or land use (e.g. residential, commercial sites) [49] variables. Moreover, it has also been applied to project the spatial extent of the dengue mosquito vector under climate change scenarios [50].

The critical systematic review of Naish et al. [4] outlined methodological issues that modeled dengue occurrence using climate variables such as study design, time periods and scale of analysis. What stands out is the diverse use of different statistical modeling techniques [51]. These studies commonly concentrate towards one statistical technique and produce combinations of meteorological factors in determining the best model for prediction. Although the review paper [4] highlighted each statistical modeling technique used, it wasn’t able to emphasize what approach can be robust or reliable in predicting and forecasting dengue incidence. For that reason, our study addresses this gap by evaluating and comparing prediction accuracy of four statistical modeling techniques in forecasting dengue incidence: General Additive Modeling (GAM), seasonal autoregressive integrated moving average (SARIMA), Random Forest (RF) and Gradient Boosting (GB). Moreover, another worth investigating is the type of dependent variables used to predict dengue incidence. Some studies would apply delayed effects or time lags of meteorological variables since it indirectly influence the occurrence of dengue. However, there are still some studies that use current observations of meteorological factors as well. Nevertheless, our study also aims to examine this area and provide basis on what kind of predictors would be well suited for prediction. Lastly, our study also explores the meteorological factors that strongly influences the dengue incidence of Metropolitan Manila based on the best model.

## Methods

### Data collection and processing

**Fig. 1:**
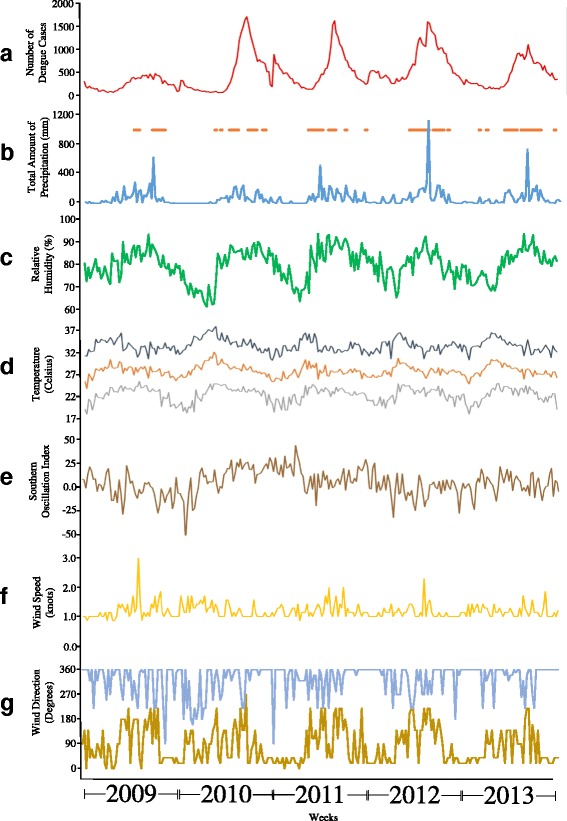
Weekly Dengue Cases and Meteorological time series of Metropolitan Manila from 2009 to 2013. (**a**) Number of dengue Cases; (**b**) Total amount of Precipitation and Presence of Flood occurrence [dots]; (**c**) Percentage of Relative Humidity; (**d**) Maximum [orange], Average [yellow] and Minimum [green] Temperatures; (**e**) Southern Oscillation Index; (**f**) Average Wind Speed and (**g**) Maximum [blue] and Minimum [green] Wind Direction

Reported dengue cases of Metropolitan Manila from January 1, 2009 until December 31, 2013 (Figure 1) were obtained from the National Epidemiology Center of the Department of Health. Most of the reported dengue cases during this period are suspected or probable cases according to standard definitions and are not laboratory confirmed. The incidence rate of dengue was calculated by dividing the total number of dengue cases by the total population of Metropolitan Manila for a particular year multiplied by a factor of 1000. The factor of 1000 refers to “per 1000 persons” in the population. The population statistics for this region were obtained from the Philippine Statistics Authority agency (www.psa.gov.ph) [52]. Since the Philippine population census was done only for years 2010 and 2015, we used the compounded population growth rate to calculate the population for years 2009, 2011, 2012, and 2013. Dengue incidence was log transformed in order to reduce the skewing of the data and to destabilize the variance which can be useful in the subsequent time series analysis.

Daily meteorological data of Metropolitan Manila from January 1, 2009 until December 31, 2013 (Figure 1) were obtained from the Philippine Atmospheric Geophysical and Astronomical Services and Administration (PAGASA). The meteorological variables include temperature (maximum, minimum and average), total precipitation, relative humidity, wind speed and wind direction. In this study, we averaged by week the daily meteorological measurements in order to conform to the weekly reported dengue cases. The average, minimum or maximum measurement of the meteorological variable for the week was used in the study for subsequent analysis. It should be noted that the wind direction variable was divided into two observations (Minimum and Maximum) because the reported values represent directionality (0°-360°).

The climate of the Philippines is highly affected by the naturally occurring El Niño/Southern Oscillation (ENSO) phenomenon that develops in the Pacific Ocean. The monitoring of ENSO in the Philippines is relatively significant because of its impact to the various sectors of the society and environment [53]. Thus, we deemed it as an important and relevant factor in the study. The Southern Oscillation Index (SOI) is considered to be the atmospheric representation of ENSO and calculated using Troup’s formula [54]. Daily calculation (January 1, 2009 until December 31, 2013) of the Southern Oscillation Index (SOI) were obtained from the Queensland Government Meteorology Bureau (http://www.longpaddock.qld.gov.au) [55] using the National Climate Centre’s revised SOI calculation based on Troup’s formula. Similar to the meteorological variables, the daily measurements were aggregated into its weekly average for subsequent analysis.

Flood occurrence in Metropolitan Manila was data-mined based on real-time public warnings of the Metropolitan Manila Development Authority (MMDA) through their social media platform, Twitter. Since flood may occur in selected parts of Metropolitan Manila because of scattered rain showers, the study considered only a flood occurrence when nearly all major roads of Metropolitan Manila are impassable due to flood. The dates of these reports were recorded and counterchecked from existing news article from credible Philippine news agencies. In the study, the presence or absence of flood during the week was done for subsequent analysis.

The entire dataset consisted of 260 observations of dengue incidence and its meteorological variables. Prior to subjecting the dataset to analysis, the dataset was divided into training and test subsets. The training subset consisted observations from the 1st morbidity week of 2009 until the last morbidity week of 2012 (*n* = 208) while the test subset consisted observations from all morbidity weeks of year 2013 (*n* = 52). The training dataset was used to build the model for the different statistical modeling techniques while the test dataset was used to validate the model.

Two types of datasets were used for each statistical modeling technique. The first dataset (MF) consisted of meteorological factors of the appropriate dengue morbidity week while the second dataset (LG) consisted only the delayed time lags of the meteorological factors. To determine the appropriate time lag for each meteorological factor, a cross correlation analysis from 0 to 25 week lags was done and the highest lag association was identified and used for the LG dataset. The number of lag weeks (*n* = 25) were identified due to cross-correlation results of past studies [18, 37, 56] using either monthly or weekly time scales.

### Modeling approaches

In our study, the natural log of dengue incidence (y) is the independent or predicted variable while the meteorological factors and its corresponding time lags are the dependent variables or predictors (x_1_, x_2_, x_3_ ….. x_n_). The statistical techniques employed are the following: (a) General Additive Modeling, (b) Seasonal autoregressive integrated moving average or SARIMA, (c) Random Forest and (d) Gradient Boosting.

### Generalized additive modeling

Generalized additive modeling (GAM) is as one approach to non-parametric regression with multiple predictors. What makes GAM unique is that it applies a “smoothing function” and it helps in capturing the predictive variables which are assumed to be non-linear in nature. GAM is often described as “data-driven” because the smoothing function automatically allows the response curve to fit for each predictor without a priori knowledge, thus generating the estimated parameters [57]. The workflow of GAM starts by first separating the predictors into sections called knots (k) then spline functions are used to fit the data into each section independently [58]. The spline function is a piecewise polynomial curve that joins these independent knots and the commonly used are the natural or B-splines. Afterwards, all functions of k are added to predict the smoothing link function. To avoid overfitting, the smooth terms of the GAM model are represented by penalized regression splines.

### Seasonal autoregressive integrated moving average with exogenous variables (SARIMAX)

The Autoregressive Integrated moving Average (ARIMA) model is considered to be the widely used forecasting model because it can project future values of a series based entirely on its own values. This approach consists of three features the Autoregressive (AR) model, Moving Average (MA) model and an initial differencing step or called Integrated (I). The first step of this approach is to meet the assumption of “stationarity” of the ARIMA model. Stationary means that the time series has no trend and its variations are around the mean and have a constant amplitude. If the original time series values are not considered stationary, then it is either transformed (i.e. natural log) or replaced the time series values with the difference (I) between the actual value and its previous value. The ARIMA model is a linear regression type of equation in which the predictors are the two remaining features, AR and MA. The AR part indicates the variable is regressed on its own lagged values while the MA corresponds to the autocorrelation error. Thus, the ARIMA model is classified as (p,d,q) model where p stands for the number autoregressive (AR) terms, d is the number of differences needed for stationarity while q is the number of lagged error in the prediction equation. Identification of the appropriate number of the AR(p) and MA(q) parameters are determined by analyzing the autocorrelation (ACF) and partial autocorrelation (PACF) function plots. The ARIMA(p,d,q) is considered to be non-seasonal model. However, if the time series has a seasonal component, it can be extended into a Seasonal ARIMA (SARIMA) where the seasonality terms are denoted as (P,D,Q)m. The P,D and Q parameters refer to the autoregressive, differencing, and moving average terms respectively while m denotes the seasonal frequency. Thus a SARIMA consists of both the non-seasonal (p,d,q) and seasonal ((P,D,Q)m) components. The SARIMA model can be further extended to include exogenous variables or regressors (SARIMA-X). The best parameters of SARIMA(p,d,q)(P,D,Q)m with exogenous variables can be selected by choosing the lowest value of Akaike Information Criterion (AIC). This model, then, can be used to forecast the dynamics of the time series.

### Random Forest and gradient boosting

Random Forest (RF) and Gradient Boosting (GB) are considered to be tree-based ensemble methods wherein it creates multiple tree sub-models and combine them to produce an improved final model. The difference between the two ensemble methods lies on the process of creating multiple tree models. RF uses the bootstrap aggregation (bagging) ensemble method in generating a large number of independent bootstrapped trees at random from the dataset. The various tree models are then aggregated or combined using the mean [59]. The final tree model estimates the importance of every predictive variable by inspecting how much the prediction error increases [60, 61]. In contrast with RF, Gradient Boosting (GB) uses stochastic gradient boosting where the model-building process is a stage-wise procedure. The task of this method is to improve the decision tree by minimizing the loss function (deviance) at each tree split. Hence, this type of ensemble method is considered to be powerful because it can enhance weak predictors by adding a predictor at a time, so that the next predictor is trained to improve the already trained ensemble therefore, improving the predictive accuracy [40].

### Implementation

All statistical modeling techniques were performed in R program version 3.3.3 [62]. Initially, Pearson’s correlation analysis was done to assess the degree of association of dengue incidence and the meteorological factors. Moreover, the correlation analysis among meteorological factors was also done to determine which meteorological factors are highly correlated with each other.

For GAM, the default parameters specified are *family = “Gaussian”*, *link = “identity”* and smoothening function using the natural spline was applied to meteorological variables except for flood occurrence. Missing values are excluded from the analysis. GAM analysis was done in a step-wise approach. Model building process was done with independent, combination and all meteorological factors and its corresponding lags. The best model was determined by Akaike Information Criterion (AIC). Determination of predictors important to the model were based on its statistical significance of the *p*-value. This statistical modeling method and process were performed using the *mgcv* package version 1.8–17 [58].

In performing SARIMAX, the *fpp* package version 0.5 [63] was employed. First, the study decided that the seasonal factor of “m” is set at 52 in order to account the annual weekly trend. Next, we applied the *xreg* function to group together the different predictor variables to be included in the model. We set the parameters for model development to be seasonal (*seasonal = TRUE*) and used the default parameters such as the AIC as the information criterion (*ic = “AIC”*) and KPSS test (*test = “kpss”*). Afterwards, the *auto.arima* function was implemented to determine the model parameters of (*p*,*d*,*q*,)(P,D,Q) with m at 52 along with predictor variables. The best model was chosen based on the lowest AIC. A Kalman filter approach was first employed to the LG dataset which contained missing values. However, the extrapolation of flood, minimum and maximum wind direction were not possible due to the nature of being categorical. Hence, the study still decided to remove the missing values found in the LG dataset similar to GAM. A stepwise approach in SARIMAX modeling building was applied to independent, combination and all meteorological factors and its corresponding lags. Moreover, identification of predictors important to the model was based on the statistical significance of the *p*-value.

The RF method is performed using *randomForest* package version 4.6.12 [64] where each model was built based on 1000 trees (*n.tree = 1000*). The *imputation* function was applied for missing values where surrogate values are generated based on weighted average of the non-missing observations. Variable importance is selected based on the number of times the variable was used for splitting, then weighted by the squared improvement to the model as a result of each split, and averaged over all trees [40]. RF generates two variable importance measures, the percentage increase of the mean square error (%IncMSE) and increase node purity (IncNodePurity).The %IncMSE is calculated as the average difference of the variables’ MSE from the original dataset and the sets of randomly permuted variables. The interpretation of a large %IncMSE value indicates a large difference from randomly permuted variables, thus, making the modeled variables important to the final model. On the other hand, IncNodePurity is a measure of the total decrease in node impurity that results from splits over that variable, averaged over all trees. Since RF shows no significance test towards which variables are important, a randomness threshold [19, 65] was used. This is calculated by dividing 100% to the number of predictor variables (MF = 10, LG = 9), hence, the study only considered a randomness threshold of above 10% in MF and 11% in LG datasets as important variables to the model.

GB models, on the other hand, comprised the following model parameters that need to be set such as learning or shrinkage rate, tree complexity and bagging factor. Learning or shrinkage rate determines the contribution of each tree to the growing model. Tree complexity determines the degree to which predictors may interact with each other and a higher value is set if more levels of interaction is needed. In the study, the interactions are fitted with a tree complexity of 5 (*tree.complexity = 5*) and learning or shrinkage rate was set at 0.001 (*learning.rate = 0.001*). The bagging factor is set at 0.5 (*bag.faction = 0.5*) as suggested by Freidman [66] and pertains that during the stage-wise approach it will randomly select 50% of the training data when the regression tree is fitted to the dependent variable. In contrast with RF, GB can accommodate missing values without the need of imputation. The GB method was performed using the *gbm* package version 2.1.3 [67]. The process of variable importance in GB is comparable with RF, however, it does not use %IncMSE nor IncNodePurity. It uses the relative contribution (%) where a higher percentage pertains to a strong relative importance of the variable. Similarly with RF that has no test of significance test on important variables, the randomness threshold was used.

### Performance evaluation

All the best models generated from each statistical modeling technique were employed to predict the dengue incidence of 2013 from the meteorological factors and its time lag of the test dataset. Afterwards, the predicted and observed values were used to calculate the root mean square error (RMSE) and mean absolute error (MAE). These two measures are commonly used to evaluate the performance of a model and determine the accuracy of regression type analysis with a continuous outcome variable [68]. The RMSE is calculated by the square root of the average of squared differences while MAE is the average over the absolute differences between predicted and actual observations. The difference between the two lies on the calculation of the error. RMSE uses the square root of the average squared errors in order to give relatively high weight to large errors. Thus, RMSE value tends to increase along with the variance of the frequency distribution of error magnitudes. On the other hand, MAE relies only to the variance of the errors without considering its magnitude. As such, MAE is considered to be an unbiased estimator while RMSE is a biased estimator due to this difference. Nevertheless, we adhere to the recommendation of the study by Chai and Draxler [68] where both measures should be used in assessing the different modeling approaches.

## Results

### Association and cross-correlation analysis

Pearson’s correlation analysis showed that 7 meteorological variables are found to be significantly associated with dengue Incidence (Table 1). These are from highest to lowest correlation; [1] relative humidity, [2] maximum temperature, [3] total rainfall, [4] mean temperature, [5] flood, [6] minimum wind direction and [7] southern oscillation index. Moreover, all climatic variables except for average wind speed, average and maximum temperature were found to have a positive association towards dengue incidence. Also, Table 1 shows the identified highest associated time lags each meteorological factor to dengue incidence. Relative humidity, rainfall and flood showed the highest association from lag weeks 4–6 respectively. Minimum, average and maximum temperatures showed the highest association from lag weeks 13–18. Lag weeks 10 and 25 were identified in minimum and maximum wind direction respectively while SOI showed highest association in lag week 20. Wind speed had correlation coefficients near zero and found to be not statistically significant, thus, this lag meteorological factor was not included in subsequent analysis.

**Table 1:** Correlation and Cross-Correlation Analysis of Meteorological Factors to Dengue Incidence

Meteorological factors	Correlation Analysis		Cross-Correlation Analysis	
	r	*p*-value	Lag week	*p*-value
Relative Humidity	0.53	0.00	4	0.00
Maximum Temperature	−0.45	0.00	18	0.00
Total Rainfall	0.31	0.00	5	0.00
Flood	0.29	0.00	6	0.00
Average Temperature	−0.29	0.00	17	0.00
Minimum Wind Direction	0.26	0.00	10	0.00
Southern Oscillation Index	0.12	0.00	20	0.00
Minimum Temperature	0.1	0.10	13	0.00
Maximum Wind Direction	0.03	0.61	25	0.29
Average Wind Speed	−0.01	0.96	–	–

Correlation analysis among meteorological factors (MF) (Additional file 1: Table S1a) showed that Average and Maximum Temperatures are highly correlated (*r* > 0.8) but not in the case of their corresponding lags (LG) (*r* < 0.8) (Additional file 1: Table S1b). We still decided to include Average Temperature for both datasets in the model development for comparative purposes and also assess in the subsequent sections if its exclusion may considerably affect the robustness of the final model (Additional file 4: Table S4).

### General additive model

**Fig. 2:**
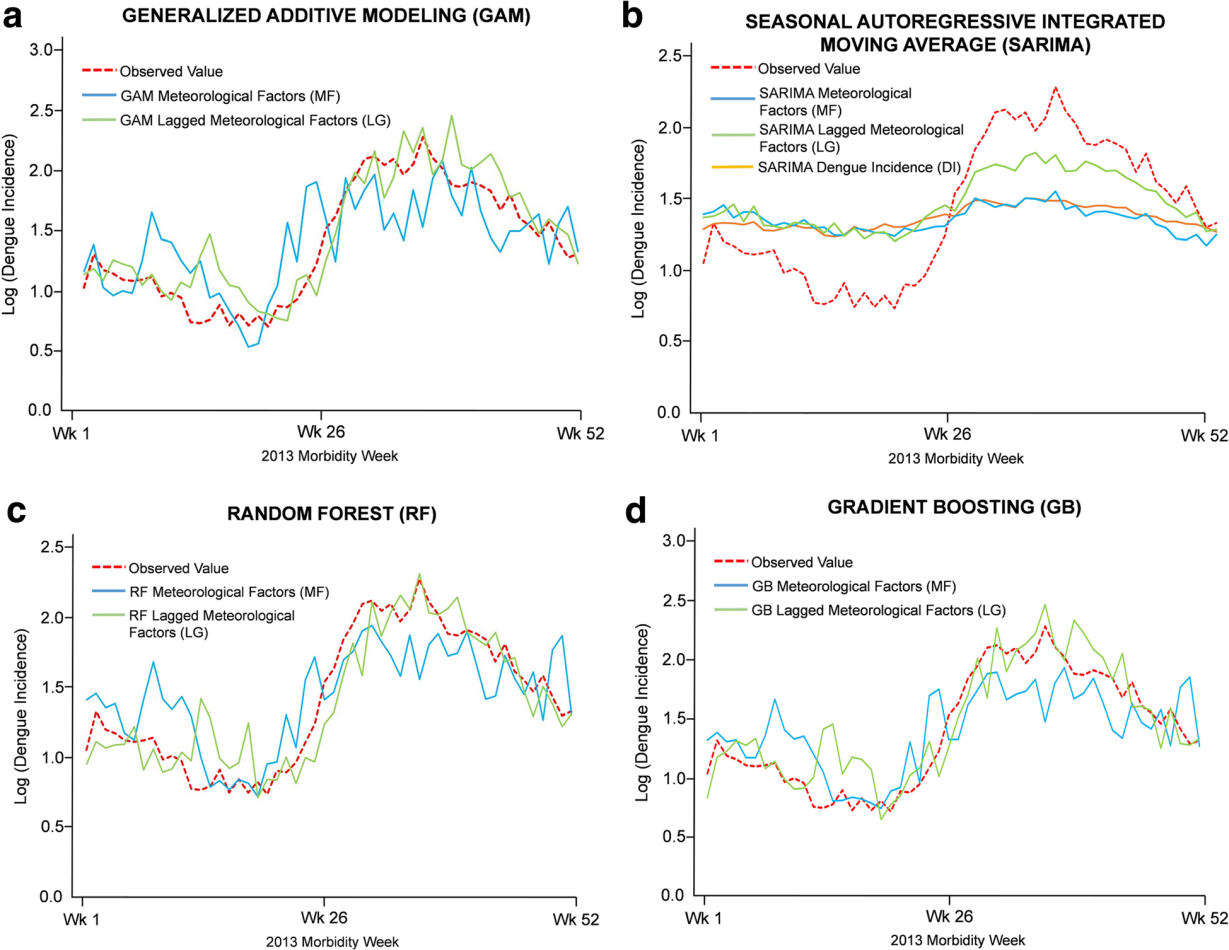
Prediction accuracy of the temporal pattern of Dengue incidence in 2013. (**a**) General Additive Modeling; (**b**) Seasonal Autoregressive Integrated Moving Average (**c**) Random Forest and (**d**) Gradient Boosting

Additional file 2: Table S2a lists the order of the different GAM models using the observed meteorological factors (MF) from the lowest to the highest AIC values. The five best models are as follows: Maximum temperature (AIC = 328.69), combination of Flood+Total Rainfall+Relative Humidity (325.41), relative humidity (324.27), Minimum+Average + Maximum Temperatures+SOI (297.34) and all- meteorological factors (286.18). Moreover, all- meteorological model (GAM-MF) resulted with the highest adjusted R^2^ (0.45) among all models used. In this best model, maximum temperature, relative humidity and SOI were considered to be the most statistically significant and important meteorological factors (Table 3) (Additional file 3: Table S3a). On the other hand, Additional file 2: Table S2b shows the order of the different GAM models using the lagged meteorological factors (LG) from the lowest to the highest AIC values. The five best models are: Maximum Temperature (265.89), Mean Temperature (255.96), Flood+Total Rainfall+Relative Humidity (238.42), Minimum+Average + Maximum Temperatures+SOI (203.63) and all- meteorological factors (145.81). The all- meteorological model (GAM-LG) was shown to be the best model with an adjusted R^2^ of 0.65 and variables that were statistically significant for the model are flood occurrence, rainfall, relative humidity, maximum temperature and SOI (Table 3) (Additional file 3: Table S3a). Since Average and Maximum Temperature were highly correlated, we examine their model quality when either one of them are excluded in model development for both datasets (MF and LG). In both MF and LG datasets, exclusion of maximum or average temperatures in the model development resulted to higher AIC values as compared to all-meteorological models (Additional file 4: Table S4a). Moreover, with the direct dependence of relative humidity with rainfall and temperature, we also explored the exclusion of this meteorological factor in model development and compared the model quality in both datasets (MF and LG). Similarly, the excluded relative humidity models were shown to generate higher AIC values than GAM-MF and GAM-LG models. Table 2 and Figure 2a show the prediction performance of the GAM-MF and GAM-LG models when using the test subset for model validation. Although both models were able to capture the dengue trend pattern, some forecast points are either overestimated or underestimated. Furthermore, the GAM-LG appears to be the better model than GAM-MF since it generated a lower RMSE and MAE values.

**Table 2:** Performance Measures of each Statistical Modeling Technique using Meteorological Factors and its Time lags in predicting the Dengue incidence of Metropolitan Manila in 2013

Statistical Modeling Technique	Datasets	Root Mean Square Error	Mean Absolute Error
General Additive Modeling (GAM)	Meteorological Factors (MF)	0.33	0.27
	Lagged MF (LG)	0.22	0.17
Seasonal Autoregressive Integrated Moving Average (SARIMAX)	Meteorological Factors (MF)	0.42	0.39
	Lagged MF (LG)	0.31	0.27
Random Forest (RF)	Meteorological Factors (MF)	0.29	0.23
	Lagged MF (LG)	0.21	0.15
Gradient Boosting (GB)	Meteorological Factors (MF)	0.30	0.24
	Lagged MF (LG)	0.23	0.17

### SARIMAX

Additional file 2 Table S2c shows the SARIMAX models using observed meteorological factors (MF) and the SARIMA model where the actual dengue incidence is used. The SARIMA model of dengue incidence is used as a baseline to compare the quality of the SARIMAX models with exogenous variables (i.e. meteorological factors). It was shown in the result that the SARIMA model of dengue incidence (SARIMA-DI) (− 144.72) have the lowest AIC value as compared to those models which included MF factors. Among the models with exogenous variables, all-meteorological factors (SARIMA-MF) showed to be the best model (− 200.67) followed by Average Wind Speed+Minimum+Maximum Wind Directions (− 201.75), Total Rainfall (− 202.86) and Flood (− 202.99). When the lagged meteorological factors are used as exogenous variables, the all-meteorological lagged factors model (SARIMA-LG) was determined to be the best model (− 125.94). It is noteworthy to mention that the SARIMA-LG resulted with the lowest AIC value as compared to the AIC values of SARIMA-DI and SARIMA-MF. Maximum, minimum temperatures, average wind speed and maximum wind direction were meteorological factors found to be significant in the SARIMA-MF model while only two variables, flood and average temperature, were found to be statistically significant in SARIMA-LG (Table 3) (Additional file 3: Table S3a). Furthermore, exclusion of average, maximum temperatures and relative humidity did not improve the model quality and performance (Additional file 4: Tables S4a and b). SARIMA-DI, SARIMA-MF and SARIMA-LG models were used to forecast the dengue incidence from the test dataset. Figure 2b shows the dengue pattern produced by the different models. It can be observed that the point forecasts are considerably away from the observed values and the dengue trend pattern is not discernable. Among the three models, SARIMA-LG model generated lowest RMSE and MAE values (Table 2).

**Table 3:** Consensus of Important Meteorological Factors (MF) and its corresponding time lags (LG) across all statistical modeling techniques

Weather Variables	Meteorological Factors (MF)			Lagged Meteorological Factors (LG)				
	GAM	SARIMAX	RF	GB	GAM	SARIMAX	RF	GB
Flood					x	x		
Rainfall			x	x	x		x	x
Relative Humidity	x		x	x	x		x	x
Minimum Temperature		x	x				x	
Average Temperature			x	x		x	x	x
Maximum Temperature	x		x	x	x		x	x
Southern Oscillation Index	x				x		x	
Wind Speed		x						
Minimum Wind Direction								
Maximum Wind Direction		x						

Note: x = identified as an important meteorological factor from each model

GAM: General Additive Modeling, SARIMAX: Seasonal Autoregressive Moving Average with Exogenous Variables, RF: Random Forest, GB: Gradient Boosting

### Random Forest and gradient boosting

**Fig. 3:**
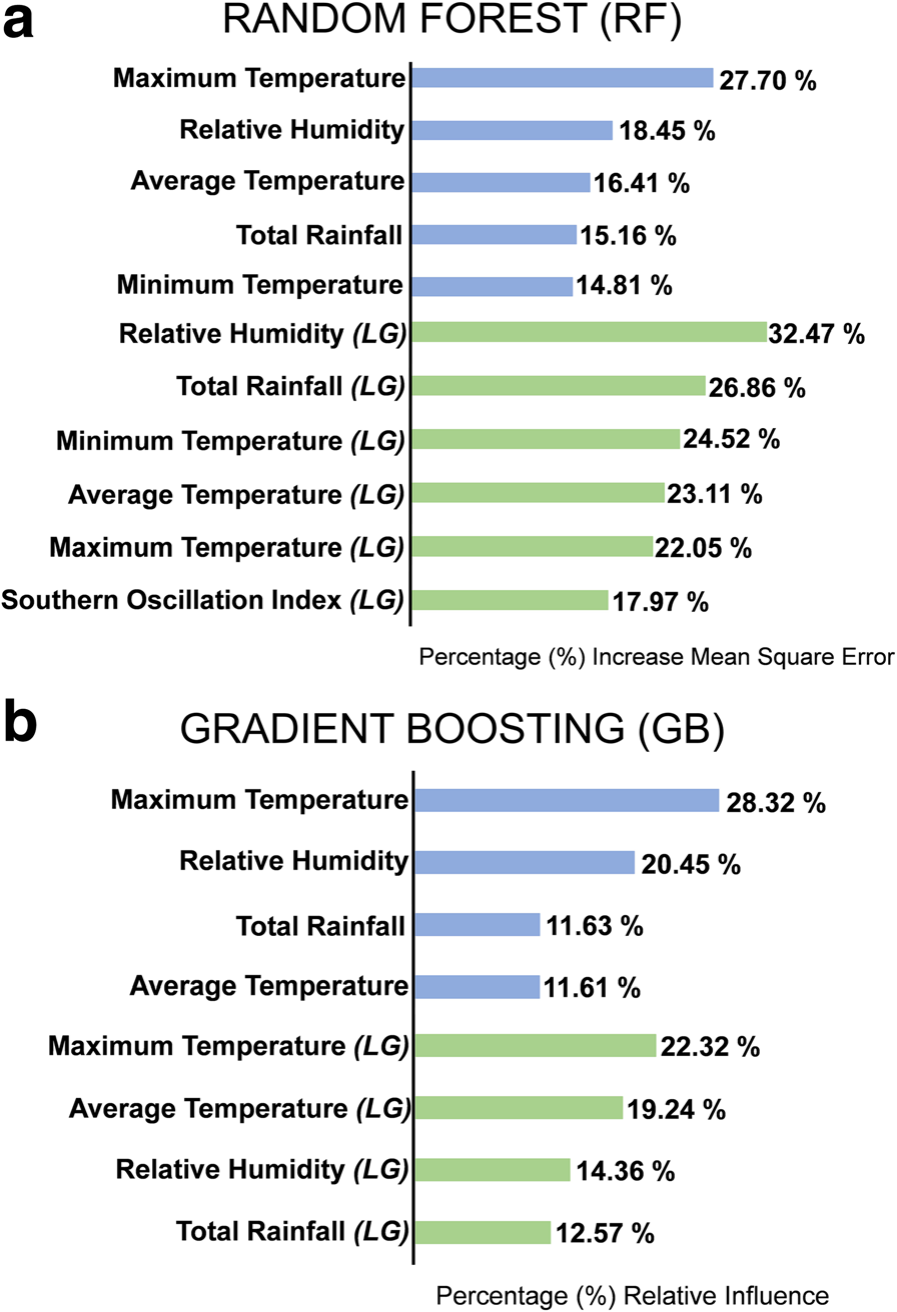
Variable importance of (**a**) Random Forest and (**b**) Gradient Boosting models. Meteorological factors (blue) and its corresponding delayed or lagged meteorological effects (LG; green). Relative contribution of Gradient Boosting models adds up to 100%

Figure 3a and Additional file 3: Table S3b shows the important variables of each RF model. For the RF meteorological factors (RF-MF) model, 5 predictors were considered above the 10% threshold; all temperatures (minimum, average and maximum), relative humidity and rainfall. On the other hand, the RF time lags of meteorological factors (RF-LG) model showed that all temperatures, relative humidity, total rainfall and southern oscillation index are above the threshold of 11%. When average, maximum temperatures and relative humidity are excluded in the model development (Additional file 4: Tables S4a and b), it did not improve the performance of the model based on its RMSE and MAE. There are instances where the removal of one predictor didn’t affect the model itself (e.g. temperatures in LG or relative humidity in MF datasets). Thus, indicating that the RF-MF and RF-LG models are still considered to be suitable. Moreover, comparing the model performance between the two types of datasets, the prediction accuracy showed that the RF-LG model produced the lower RMSE and MAE values than RF-LG model (Table 2). Figure 2c shows that that two models were able to capture the dengue trend pattern of 2013. However, majority of the predictive values of RF-MF were either overestimated or underestimated.

Figure 3b and Additional file 3: Table S3b shows the important variables of each GB model. In the GB meteorological factors model (GB-MF), four factors were only considered above the 10% threshold; relative humidity, rainfall, average and maximum temperature. On the other hand, the GB lagged meteorological (GB-LG) model determined 5 lagged meteorological factors above the 11% threshold; relative humidity, rainfall, southern oscillation index, average and maximum temperatures. GB-MF and GB-LG are still considered the suitable models even though maximum, average temperatures and relative humidity are excluded in the model development (Additional file 4: Tables S4a and b). Furthermore, the prediction accuracy of the GB-LG model generated lower RMSE and MAE values than the GB-MF model (Table 2). All models were able to capture the dengue trend pattern of 2013 however majority of the predictive values were either overestimated or underestimated (Fig. 2d).

### Comparison and evaluation of model performance across all statistical modeling techniques

Among the statistical modeling techniques, RF showed the lowest RMSE and MAE values across all types of datasets (Table 2). Moreover, it was also shown that LG datasets provided the lowest values across the different statistical modeling techniques. The RF-LG model showed to be the best model as evidence from the performance measures and the pattern of the predictive values against the observed values. Table 3 shows the variable importance of the four statistical modeling technique from each type of dataset. It is clear that no factor is commonly found in all techniques except for relative humidity and maximum temperature. The two factors are determined important by GAM, RF and GB in both datasets (MF and LG). Furthermore, rainfall and average temperature appears to be also mutually important too in the LG datasets.

## Discussion

### Comparison of the statistical modeling techniques and datasets

Overall, the two machine learning methods (MLMs), Random Forest and Gradient Boosting outperformed the two conventional statistical modeling techniques in predicting the temporal dengue incidence of Metro Manila in 2013. The findings of our study are consistent with other literature in showing the exceptional performance of these methods over classical methods [45, 69, 70]. MLMs have several advantages as compared to conventional statistical techniques. One such case is the robustness towards outliers. Parametric statistics such as means, standard deviation and correlations are highly sensitive to outliers. Thus, conventional statistical procedures that are based on these parametric statistics may severely influence the final model especially on the predicted responses. The reason why tree-based ensemble methods are robust to outliers because of its fundamental process of splitting and partitioning the dataset. The split can occur based on the proportion of samples within a certain range. Therefore, it is known that outliers would not affect the splitting rule at each node of the tree model. GB was the only method to handle and accommodate missing values. Moreover, imputation of missing values was needed for RF in order to implement model development. This goes the same with SARIMA however, during the imputation process using Kalman filter approach, extrapolation of categorical meteorological factors (e.g. Flood) were not possible. GAM, on the other hand, may only use complete datasets, thus the removal of observations with incomplete entries was needed. Thus, only selected MLMs may have the capability to accommodate missing values.

Among all statistical methods used in our study, Random Forest has shown to be the best method in predicting the temporal dengue incidence of Metropolitan Manila. This is consistent with other studies where they find this method as robust and accurate [71–73]. Although there are studies which shows GB to be superior from RF, the use of these methods may generate mixed results [74] when applied to different types of dataset. It is highly recommended that applying these methods should compare both results in order to select the best model for prediction as demonstrated by this study. The observed lower RMSE and MAE values in RF can be explained towards how both algorithmic models tackles the bias-variance dilemma [75]. Generally, GB’s algorithm is tasked to reduce bias by producing shallow trees while RF reduces variance by generating fully grown decision trees. Moreover, the GB process uses each tree model as its basis to correct errors in its stage-wise approach, thus, producing a higher variance as compared to RF.

Our study has clearly demonstrated that the use of delayed effects or time lags is suitable towards the prediction of dengue incidence. These time lags account for the potential delays in the time which weather affects both the mosquito vector and virus. Since our analysis explored the influence of meteorological factors towards the disease occurrence, the observed time lags may potentially explain the dengue temporal variability in Metropolitan Manila. Time lags are appropriate in model development for dengue due to the nature of its disease dynamics where it starts with mosquito development, mosquito acquisition and amplification of the virus, seeking host behavior of mosquitoes and virus incubation in humans leading to disease outbreak [76, 77]. The impact of time lag effect on dengue incidence has been investigated by numerous studies [16, 37, 78, 79] (with varying lags observed for each meteorological factor. With this, it is clearly appropriate and applicable the use of time lags in predicting dengue incidence especially when using meteorological factors.

It should be known that our study is inherent with certain limitations. First, the study specifies only one location in the Philippines which is Metropolitan Manila. The application of the results may only be good for this area but may change to some extent when applied to other regions in the Philippines. Different regions may possess diverse types of landscapes (i.e. urban or rural) or experience atypical ENSO effects that can feature different annual climatological cycles as well as meteorological profiles. Thus, leading to other meteorological factors or teleconnections besides ENSO to be important as compared to the results of this study. Another that needs to be considered is the multifacet nature of dengue disease dynamics where the study limited itself to a selected ecological factor. Other factors such as biological (e.g. mosquito surveillance and serological surveys) and sociological (human movement, health services) aspects may be added in the future along with meteorological variables. It is noteworthy to mention that the Philippines had started to use the approved dengue vaccine (Dengvaxia®) [80] in high dengue endemic areas such as Metropolitan Manila. It is expected that dengue morbidity will lower as compared to previous years, thus, affecting the prediction nature of the models used in the study. It is essential that such event should be included in future endeavors of dengue risk modeling. Nonetheless, the results of the study provide valuable insights when employing different modeling approaches. This may serve as a guide especially for those who are embarking to model factors that are linked with dengue disease using recent modeling techniques such as MLMs.

### Important meteorological factors in predicting dengue incidence

The impact of meteorological factors towards dengue incidence has been widely studied. The most common meteorological factors associated with dengue incidence are rain, temperature and relative humidity [81–84]. Following the factors deemed important by the best model, RF-LG, rainfall, relative humidity, temperature, and SOI were selected. These findings are consistent with previous results [28, 33] except for relative humidity. In our study, we find that a positive correlation of rainfall to dengue incidence. Moreover, the annual increasing trend of dengue cases in the Philippines, especially in the study area, happens during the rainy season (June – October) of the Philippines. The presence of water bodies plays a big role in the abundance and development of the dengue vector mosquito. *Aedes aegypti* mosquitoes are holometabolous insects wherein it relies on water bodies to complete its general life cycle of about two weeks under ideal conditions [85]. Hence, the presence of rainfall provides breeding habitats and opportunities for these mosquito vectors to proliferate or become abundant in the environment [86]. However, there is still a debate on whether this factor is considered as the sole basis of explaining the weekly occurrence of dengue. In Singapore, for example, rainfall was determined not highly influential towards the temporal pattern of dengue cases [87]. The likely impact of rainfall may be restricted due to the fact that majority of the breeding sites of *Ae aegpyti* are found indoors. This may possibly be one of the reasons why dengue is incessant in Metropolitan Manila especially during the non-rainy months throughout the year.

With the Philippines being a tropical country, Metropolitan Manila’s temperature annual do not vary as much. The study area has an average annual temperature of 27.91 °C with an average minimum temperature of 22.51 °C and an average maximum temperature of 33.60 °C during 2009–2013. Our correlation analysis found a positive association in minimum temperature while a negative association in average and maximum temperature. Thus pertaining that high dengue occurrence may occur in temperatures between 23 °C -28 °C in Metropolitan Manila. The role of temperature has been considered to be the driving factor for mosquito development, thus leading to its abundance. Several studies [88–91] have conducted experiments to ascertain the optimal temperature for the development and survival of the vector. Because of such, this had led to the basis that these mosquitoes can spatially expand their habitat under climate-based scenarios. Additionally, temperature regulation in breeding containers was determined to be a factor towards the developmental rate of the dengue mosquito [92]. Changes in temperature not only affect the biology and ecology of the mosquito but also the dengue virus. There is compelling evidence that changes in temperature is associated in the replication, maturation and infective periods of the virus in mosquitoes [37, 93].

What is noteworthy in our findings is the importance of relative humidity. In our correlation analysis, it was ranked with the highest association among all meteorological variables. Furthermore, relative humidity appears to be an important meteorological factor in most of the statistical techniques employed in the study. Although there is a direct dependence of relative humidity towards temperature and rainfall, our study demonstrated that the omission of such affect the model quality and performance of the different models. Thus, indicating its non-dependency towards rainfall or temperature. Relative humidity was neither emphasized nor highlighted by previous Philippine studies [28, 33]. There is a great deal of emphasis of this meteorological factor linking the biology and population dynamics of *Ae. aegypti*. Relative humidity had been claimed to be a consistent and substantial factor that provides a suitable condition for the development and survival of the mosquito vector [94]. In Thailand, relative humidity is deemed to be a determinant in the egg development and adult population size of the mosquito vector [95]. It was also observed in Texas that there was an increase in the hatching rate of *Ae. aegypti* eggs whenever there is an increase in relative humidity [96]. Moreover, relative humidity is highlighted to be a crucial factor in affecting the life patterns of the mosquito dengue vectors such as mating, oviposition, and seeking host pattern [97] which are necessary for increasing dengue transmission.

Another notable observation in the results is that no single factor was found to be important across all models. However, relative humidity and maximum temperature appear to be the common important factors found in the models of GAM, RF and GB in both datasets. Additionally, it appears that conventional modeling techniques (e.g. SARIMAX) tend to obtain different statistical significant factors when a different dataset is applied. Unlike MLMs, the important factors identified were nearly the same in MF and LG datasets. It is the intention of the study to capture common meteorological factor/s when different statistical modeling techniques are applied. This pertains that the factor is really robust amidst on how it is used in model development. Therefore, our study may only infer the importance of the two meteorological factors because of its frequent occurrence across modeling techniques. Moreover, our study would also like to emphasize that it does not challenge nor invalidate the importance of other meteorological factors such as rainfall or flood. We acknowledge these meteorological factors to be highly important due to its link in dengue disease dynamics. The importance of relative humidity was indeed a remarkable outcome in the study, thus broadening our current perspective and understanding on its influence in dengue disease dynamics of Metropolitan Manila. Lastly, our study is a clear demonstration of how different modeling approaches may tend to yield diverse meteorological factors as important predictors. Thus, it is imperative to be mindful on what kind of datasets to use (e.g. lag factors) or the application of a modeling approach in order to present a meaningful interpretation. It is recommended to future researchers to apply the same approach employed by the study in order to gain valuable insights not only in its methodological aspect but to other potential effects of meteorological factors to dengue occurrence.

## Conclusion

Our study has revealed that Tree-based Machine Learning methods (RF and GB) performed well in predicting the temporal pattern of dengue Incidence of Metropolitan Manila as compared to conventional statistical modeling techniques (GAM and SARIMAX). Our study also highlighted that the use of delayed effects or time lags (LG) of each meteorological factor are more appropriate in predicting dengue incidence. With this, our approach may serve as a basis towards choosing an appropriate modeling technique to be used in adopting an early outbreak warning system. It is further recommended that other factors such as temporal vector abundance and virus epidemiology should be included in order to broaden the scope of understanding and perspective of dengue disease.

Relative humidity was deemed by our study to be also an important variable in the RF-LG model along with rainfall and temperature. This meteorological factor was not only found from the best model but from other models as well either on the top two or three most important variable. Such finding is noteworthy because it provides a broader perspective and understanding on how meteorological variables tend to influence the temporal pattern of disease occurrence in Metropolitan Manila. This is worth investigating for future endeavors since no study has ever been conducted yet in exploring this meteorological factor to dengue disease in the Philippines.

## Additional files

Additional file 1: **Table S1a.** Correlation Analysis among Meteorological factors (MF). **Table S1b.** Correlation Analysis among Lagged Meteorological factors (LG). (DOCX 23 kb)

Additional file 2: **Table S2a.** Adjusted R-square and Akaike Information Criterion of Meteorological Factors (MF) using Generalized Additive Modeling (GAM). **Table S2b.** Adjusted R-square and Akaike Information Criterion of Lagged Meteorological Factors (LG) using Generalized Additive Modeling (GAM). **Table S2c.** Model Quality of Seasonal Autoregressive Integrated Moving Average (SARIMA) Model ((p,d,q)(P,D,Q)m) with Meteorological Factors (MF) as Exogenous Variables based on the Akaike Information Criterion. **Table S2d.** Model Quality of Seasonal Autoregressive Integrated Moving Average (SARIMA) Model ((p,d,q)(P,D,Q)m) with Lagged Meteorological Factors (LG) as Exogenous Variables based on the Akaike Information Criterion (DOCX 21 kb)

Additional file 3: **Table S3a.** Statistical significant Meteorological (MF) and its Lagged (LG) Factors in General Additive Modeling (GAM) and Seasonal Autoregressive Integrated Moving Average with Exogenous Variables (SARIMAX). **Table S3b.** Variable Importance of Random Forest and Gradient Boosting in Meteorological factors (MF) and its corresponding lags (LG). (DOCX 21 kb)

Additional file 4: **Table S4a.** Comparison of Model Quality and Performance in Meteorological Factors (MF) dataset when Maximum, Average Temperatures and Relative Humidity are excluded in the Model Development. **Table S4b.** Comparison of Model Quality and Performance in Lagged Meteorological Factors (LG) dataset when Maximum, Average Temperatures and Relative Humidity are excluded in the Model Development. (DOCX 13 kb)

## Abbreviations

ACF: autocorrelation function
DOH: Department of Health - Philippines
GAM: Generalized Additive Modeling
GB: Gradient Boosting
MMDA: Metropolitan Manila Development Authority
NEC: National Epidemiology Center – DOH, Philippines
PACF: partial autocorrelation function
PAGASA: Philippine Atmospheric Geophysical and Astronomical Services and Administration
RF: Random Forest
SARIMAX: Seasonal Autoregressive Integrated Moving Average with Exogenous Variables
SOI: Southern Oscillation Index
WHO: World Health Organization

## References

[1] Martina BE, Koraka P, Osterhaus AD (2009). Dengue virus pathogenesis: an integrated view. Clin Microbiol Rev.

[2] World Health Organization. Dengue and severe dengue. Fact Sheet. http://www.who.int/mediacentre/factsheets/fs117/en/index/html (2017). Accessed 15 Jan 2017.

[3] Tana S, Abeyewickreme W, Arunachalam N, Espino F, Kittayapong P, Wai KT, Horstick O, Sommerfeld J. Eco-Bio-Social research on dengue in Asia: general principles and a case study.

[4] Naish S, Dale P, Mackenzie JS, McBride J, Mengersen K, Tong S (2014). Climate change and dengue: a critical and systematic review of quantitative modelling approaches. BMC Infect Dis.

[5] Chowell G, Sanchez F (2006). Climate-based descriptive models ofdengue fever: the 2002 epidemic in Colima, Mexico. J Environ Health.

[6] Hii YL, Rocklov J, Ng N, Tang CS, Pang FY, Sauerborn R (2009). Climate variability and increase in intensity and magnitude of dengue incidence in Singapore. Glob Health Action.

[7] Jetten TH, Focks DA (1997). Potential changes in the distribution of dengue transmission under climate warming. The American journal of tropical medicine and hygiene.

[8] Morin CW, Comrie AC, Ernst K (2013). Climate and dengue transmission: evidence and implications. Environ Health Perspect.

[9] Kearney M, Porter WP, Williams C, Ritchie S, Hoffmann AA (2009). Integrating biophysical models and evolutionary theory to predict climatic impacts on species’ ranges: the dengue mosquito *Aedes aegypti* in Australia. Funct Ecol.

[10] Hopp MJ, Foley JA (2001). Global-scale relationships between climate and the dengue fever vector, *Aedes aegypti*. Clim Chang.

[11] Peterson AT, Martínez-Campos C, Nakazawa Y, Martínez-Meyer E (2005). Time-specific ecological niche modeling predicts spatial dynamics of vector insects and human dengue cases. Trans R Soc Trop Med Hyg.

[12] Lindsay M, Mackenzie J, Curson P, Guest C, Jackson E (1997). Vector-borne viral diseases and climate change in the Australian region: major concerns and the public health response. Climate changes and human health in the Asia Pacific region. Canberra: Australian medical association and Greenpeace international.

[13] Gratz NG (1999). Emerging and resurging vector-borne disease. Annu Rev Entomol.

[14] Hales S, de Wet N, Maindonaid J, Woodward A (2002). Potential effect of population and climatic changes on global distribution of dengue fever: an empirical model. Lancet.

[15] Mellor PS, Leake CJ (2000). Climatic and geographic influences on arboviral infections and vectors. Rev Sci Tech.

[16] Depradine CA, Lovell EH (2004). Climatological variables and the incidence of dengue fever in Barbados. Int J Environ Health Res.

[17] Yasuoka J, Levins R (2007). Ecology of vector mosquitoes in Sri Lanka--suggestions for future mosquito control in rice ecosystems. Southeast Asian J Trop Med Public Health.

[18] Rosa-Freitas MG, Schreiber KV, Tsouris P, Weimann ET, Luitgards-Moura JF (2006). Associations between dengue and combinations of weather factors in a city in the Brazilian Amazon. Rev Panam Salud Publica.

[19] Lowe R, Bailey TC, Stephenson DB, Graham RJ, Coelho CA, Carvalho MS, Barcellos C (2011). Spatio-temporal modelling of climate-sensitive disease risk: towards an early warning system for dengue in Brazil. Comput Geosci.

[20] Lowe R, Bailey TC, Stephenson DB, Jupp TE, Graham RJ, Barcellos C, Carvalho MS (2013). The development of an early warning system for climate-sensitive disease risk with a focus on dengue epidemics in Southeast Brazil. Stat Med.

[21] Cheong YL, Burkart K, Leitão PJ, Lakes T (2013). Assessing weather effects on dengue disease in Malaysia. Int J Environ Res Public Health.

[22] Foo LC, Lim TW, Lee HL, Fang R (1985). Rainfall, abundance of *Aedes aegypti* and dengue infection in Selangor, Malaysia. Southeast Asian J Trop Med Public Health.

[23] Hii YL, Zaki RA, Aghamohammadi N, Rocklöv J (2016). Research on climate and dengue in Malaysia: a systematic review. Current environmental health reports.

[24] Promprou S, Jaroensutasinee M (2005). Jaroensuta. Climatic factors affecting dengue haemorrhagic fever incidence in southern Thailand Dengue Bulletin.

[25] Wongkoon S, Jaroensutasinee M, Jaroensutasinee K, Preechaporn W, Chumkiew S (2007). Larval occurrence and climatic factors affecting DHF incidence in Samui Islands, Thailand. World Acad Sci Eng Technol.

[26] Pham HV, Doan HT, Phan TT, Minh NN (2011). Ecological factors associated with dengue fever in a central highlands province, Vietnam. BMC Infect Dis.

[27] Lee HS, Nguyen-Viet H, Nam VS, Lee M, Won S, Duc PP, Grace D (2017). Seasonal patterns of dengue fever and associated climate factors in 4 provinces in Vietnam from 1994 to 2013. BMC Infect Dis.

[28] Sia Su GL (2008). Correlation of climatic factors and dengue incidence in metro manila, Philippines. AMBIO: A Journal of the Human Environment.

[29] Hii YL, Rocklo’v J, Wall S, Ng LC, Tang CS (2012). Optimal lead time for dengue forecast. PLoS Negl Trop Dis.

[30] Hii YL, Zhu H, Ng N, Ng L, Rocklo’v J (2012). Forecast of dengue incidence using temperature and rainfall. PLoS Negl Trop Dis.

[31] Earnest A, Tan SB, Wilder-Smith A (2012). Meteorological factors and El Nino southern oscillation are independently associated with dengue infections. Epidemiology & Infection.

[32] Ramadona AL, Lazuardi L, Hii YL, Holmner Å, Kusnanto H, Rocklöv J (2016). Prediction of dengue outbreaks based on disease surveillance and meteorological data. PLoS One.

[33] Arcenas AL (2016). (DP 2016-01) climate change, dengue and the economy: ascertaining the link between dengue and climatic conditions. UPSE Discussion Papers.

[34] Chuang TW, Chaves LF, Chen PJ (2017). Effects of local and regional climatic fluctuations on dengue outbreaks in southern Taiwan. PLoS One.

[35] Jbilou J, El Adlouni S. Generalized additive models in environmental health: a literature review. In Novel Approaches and Their Applications in Risk Assessment. 2012. Available from: http://www.intechopen.com/books/novel-approaches-and-theirapplications-in-risk-assessment/generalized-additive-models-in-environmental-health-a-review-of-litterature.

[36] Yang L, Qin G, Zhao N, Wang C, Song G (2012). Using a generalized additive model with autoregressive terms to study the effects of daily temperature on mortality. BMC Med Res Methodol.

[37] Wu PC, Guo HR, Lung SC, Lin CY, Su HJ (2007). Weather as an effective predictor for occurrence of dengue fever in Taiwan. Acta Trop.

[38] Abeare SM (2009). Comparisons of boosted regression tree, GLM and GAM performance in the standardization of yellowfin tuna catch-rate data from the Gulf of Mexico Lonline fishery.

[39] Olden JD, Lawler JJ, Poff NL (2008). Machine learning methods without tears: a primer for ecologists. Q Rev Biol.

[40] Elith J, Leathwick JR, Hastie T (2008). A working guide to boosted regression trees. J Anim Ecol.

[41] Kane MJ, Price N, Scotch M, Rabinowitz P (2014). Comparison of ARIMA and random Forest time series models for prediction of avian influenza H5N1 outbreaks. BMC bioinformatics.

[42] Ruiz MO, Chaves LF, Hamer GL, Sun T, Brown WM, Walker ED (2010). Local impact of temperature and precipitation on West Nile virus infection in Culex species mosquitoes in Northeast Illinois, USA. Parasit Vectors.

[43] Deconinck E, Hancock T, Coomans D, Massart DL, Vander Heyden Y (2005). Classification of drugs in absorption classes using the classification and regression trees (CART) methodology. J Pharm Biomed Anal.

[44] Coutts SR, Yokomizo H (2014). Meta-models as a straightforward approach to the sensitivity analysis of complex models. Popul Ecol.

[45] Ismail R, Mutanga OA (2010). Comparison of regression tree ensembles: predicting Sirex noctilio induced water stress in Pinus patula forests of KwaZulu-Natal, South Africa. Int J Appl Earth Obs Geoinf.

[46] Fathima A, Manimegalai D (2012). Predictive analysis for the arbovirus-dengue using svm classification. International Journal of Engineering and Technology.

[47] Brasier AR, Zhao Y, Wiktorowicz JE, Spratt HM, Nascimento EJ, Cordeiro MT, Soman KV, Ju H, Recinos A, Stafford S, Wu Z (2015). Molecular classification of outcomes from dengue virus-3 infections. J Clin Virol.

[48] Soonthornphisaj N (2016). Knowledge discovery on dengue fever using data mining techniques. Journal of Thai Interdisciplinary Research.

[49] Cheong YL, Leitão PJ, Lakes T (2014). Assessment of land use factors associated with dengue cases in Malaysia using boosted regression trees. Spatial and spatio-temporal epidemiology.

[50] Bhatt S, Gething PW, Brady OJ, Messina JP, Farlow AW, Moyes CL, Drake JM, Brownstein JS, Hoen AG, Sankoh O, Myers MF (2013). The global distribution and burden of dengue. Nature.

[51] Chaves LF, Pascual M (2007). Comparing models for early warning systems of neglected tropical diseases. PLoS Negl Trop Dis.

[52] Philippine Statistics Authority: Population and Housing. http://psa.gov.ph/ (2016). Accessed on Jun 2016.

[53] Hilario F, de Guzman R, Ortega D, Hayman P, Alexander B (2009). El Niño southern oscillation in the Philippines: impacts, forecasts, and risk management. Philippine Journal of Development.

[54] Troup AJ (1965). The ‘southern oscillation’. Q J R Meteorol Soc.

[55] Queensland Government Meteorology Bureau: Southern Oscillation Index. http://www.longpaddock.qld.gov.au (2016) Accessed on October 2016.

[56] Chen SC, Liao CM, Chio CP, Chou HH, You SH, Cheng YH (2010). Lagged temperature effect with mosquito transmission potential explains dengue variability in southern Taiwan: insights from a statistical analysis. Sci Total Environ.

[57] Wood SN, Augustin NH (2002). GAMs with integrated model selection using penalized regression splines and applications to environmental modelling. Ecol Model.

[58] Wood S (2006). Generalized additive models: an introduction with R.

[59] Pino-Mejías R, Cubiles-de-la-Vega MD, Anaya-Romero M, Pascual-Acosta A, Jordán-López A, Bellinfante-Crocci N. Predicting the potential habitat of oaks with data mining models.

[60] Liaw A, Wiener M (2002). Classification and regression by randomForest. R news.

[61] Catani F, Lagomarsino D, Segoni S, Tofani V (2013). Landslide susceptibility estimation by random forests technique: sensitivity and scaling issues. Nat Hazards Earth Syst Sci.

[62] R Development Core Team (2016). R: a language and environment for statistical computing. R foundation for statistical computing.

[63] Hyndman RJ, Athanasopoulos G. Forecasting: principles and practice. OTexts, 2014. The book is freely available as an online book at www. otexts. org/fpp. Alternatively, a print version is available: ISBN. 2014;987507109.

[64] Breiman, L., Cutler, A., Liaw, A., and Wiener M. randomForest: Breiman and Cutler's random forests for classification and regression. R package version 4.6-12. Available at CRAN. R-project. Org/package= randomForest (2016). Accessed June 2016.

[65] Müller D, Leitão PJ, Sikor T (2013). Comparing the determinants of cropland abandonment in Albania and Romania using boosted regression trees. Agric Syst.

[66] Friedman JH. Greedy function approximation: a gradient boosting machine. Ann Stat. 2001 Oct 1:1189–232.

[67] Ridgeway G. Gbm: generalized boosted regression models. R package version 2.1.3. Available at CRAN. R-project. Org/package= gbm (2016). Accessed June 2016.

[68] Chai T, Draxler RR (2014). Root mean square error (RMSE) or mean absolute error (MAE)?. Geoscientific Model Development Discussions.

[69] Oppel S, Meirinho A, Ramirez I, Gardner B, O'Connell AF, Miller PI, Louzao M (2012). Comparison of five modelling techniques to predict the spatial distribution and abundance of seabirds. Biol Conserv.

[70] Naghibi SA, Pourghasemi HR, Pourtaghi ZS, Rezaei A (2015). Groundwater qanat potential mapping using frequency ratio and Shannon's entropy models in the Moghan watershed, Iran. Earth Sci Inf.

[71] Rahmati O, Pourghasemi HR, Melesse AM (2016). Application of GIS-based data driven random forest and maximum entropy models for groundwater potential mapping: a case study at Mehran region, Iran. Catena.

[72] Carranza EJ, Laborte AG (2015). Data-driven predictive mapping of gold prospectivity, Baguio district, Philippines: application of random forests algorithm. Ore Geol Rev.

[73] Hamza M, Larocque D (2005). An empirical comparison of ensemble methods based on classification trees. J Stat Comput Simul.

[74] Breiman L (2001). Random forests. Mach Learn.

[75] Geman S, Bienenstock E, Doursat R (1992). Neural networks and the bias/variance dilemma. Neural Comput.

[76] Gharbi M, Quenel P, Gustave J, Cassadou S, La Ruche G, Girdary L, Marrama L: Time series analysis of dengue incidence in Guadeloupe, French West Indies: forecasting models using climate variables as predictors. BMC Infect Dis. 2011, 11: 166–10.1186/1471-2334-11-166. 10.1186/1471-2334-11-166 PMC3128053 21658238

[77] Dhiman RC, Pahwa S, Dhillon GP, Dash AP (2010). Climate change and threat of vector-borne diseases in India: are we prepared?. Parasitol Res.

[78] Keating J (2001). An investigation into the cyclical incidence of dengue fever. Soc Sci Med.

[79] Arcari P, Tapper N, Pfueller S (2007). Regional variability in relationships between climate and dengue/DHF in Indonesia. Singap J Trop Geogr.

[80] Sanofi Pasteur. Sanofi Pasteur’s dengue vaccine approved in the Philippines. Available at: http://www.sanofipasteur.com/en/articles/sanofi-pasteur-dengue-vaccine-approved-in-thephilippines.aspx (2016). Accessed 26 Oct 2017.

[81] Tong S (2008). Impact of Climate Change on Vectorborne Disease: What are the Early Signs so Far?. Epidemiology.

[82] Shuman EK (2010). Global climate change and infectious diseases. N Engl J Med.

[83] Banu S, Guo Y, Hu W, Dale P, Mackenzie JS, Mengersen K, Tong S. Impacts of El Ni?O southern oscillation and Indian Ocean dipole on dengue incidence in Bangladesh. Sci Rep. 2015;5 10.1038/srep16105 PMC4633589 26537857

[84] Limper M, Thai KTD, Gerstenbluth I, Osterhaus ADME, Duits AJ, van Gorp ECM (2016). Climate factors as important determinants of dengue incidence in Curaçao. Zoonoses Public Health.

[85] Reiter P (2010). Yellow fever and dengue: a threat to Europe?. EuroSurveill.

[86] Murray NE, Quam MB, Wilder-Smith A (2013). Epidemiology of dengue: past, present and future prospects. Clinical epidemiology.

[87] Xu HY, Fu X, Lee LK, Ma S, Goh KT, Wong J, Habibullah MS, Lee GK, Lim TK, Tambyah PA, Lim CL (2014). Statistical modeling reveals the effect of absolute humidity on dengue in Singapore. PLoS Negl Trop Dis.

[88] Tun-Lin W, Burkot TR, Kay BH (2000). Effects of temperature and larval diet on development rates and survival of the dengue vector *Aedes aegypti* in North Queensland, Australia. Med Vet Entomol.

[89] Yang HM, Macoris MLG, Galvani KC, Andrighetti MTM, Wanderley DMV (2009). Assessing the effects of temperature on the population of *Aedes aegypti*, the vector of dengue. Epidemiol Infect.

[90] Lambrechts L, Paaijmans KP, Fansiri T, Carrington LB, Kramer LD, Thomas MB, Scott TW (2011). Impact of daily temperature fluctuations on dengue virus transmission by *Aedes aegypti*. Proc Natl Acad Sci.

[91] Eisen L, García-Rejón JE, Gómez-Carro S, Vázquez MD, Keefe TJ, Beaty BJ, Loroño-Pino MA (2014). Temporal correlations between mosquito-based dengue virus surveillance measures or indoor mosquito abundance and dengue case numbers in Merida City, Mexico. J Med Entomol.

[92] Almanzor BL, Ho HT, Carvajal TM (2016). Ecdysis period and rate deviations of dengue mosquito vector, *Aedes aegypti* reared in different artificial water-holding containers. Journal of vector borne diseases.

[93] Fan J, Wei W, Bai Z, Fan C, Li S, Liu Q, Yang K (2014). A systematic review and meta-analysis of dengue risk with temperature change. Int J Environ Res Public Health.

[94] Descloux E, Mangeas M, Menkes CE, Lengaigne M, Leroy A, Tehei T, Guillaumot L, Teurlai M, Gourinat AC, Benzler J, Pfannstiel A (2012). Climate-based models for understanding and forecasting dengue epidemics. PLoS Negl Trop Dis.

[95] Vargas RE, Ya-umphan P, Phumala-Morales N, Komalamisra N, Dujardin JP. Climate associated size and shape changes in *Aedes aegypti* (Diptera: Culicidae) populations from Thailand. Infect Genet Evol 2010 May 31;10(4):580–2015. 10.1016/j.meegid.2010.01.004 20123039

[96] Dickerson CZ (2007). The effects of temperature and humidity on the eggs of *Aedes aegypti* (L.) and *Aedes albopictus* (Skuse) in Texas. Texas a&M university.

[97] Wu JY, Lun ZR, James AA, Chen XG (2010). Dengue fever in mainland China. Am J Trop Med Hyg.

