# Supplementary material for: Machine learning methods reveal the temporal pattern of dengue incidence using meteorological factors in metropolitan Manila, Philippines
Source: BMC Infect Dis. 2018 Apr 17;18:183. doi: 10.1186/s12879-018-3066-0 (PMC5905126; doi:10.1186/s12879-018-3066-0)
Supplement: Supplementary file 1 — Table S1a. Correlation Analysis among Meteorological factors (MF). Table S1b. Correlation Analysis among Lagged Meteorological factors (LG). (DOCX 23 kb) [file 12879_2018_3066_MOESM1_ESM.docx]

**Table S1a. Correlation Analysis among Meteorological factors (MF)**

| **Meteorological Factors** | | **A** | **B** | **C** | **D** | **E** | **F** | **G** | **H** | **I** | **J** |
| --- | --- | --- | --- | --- | --- | --- | --- | --- | --- | --- | --- |
| **A** | **Flood**  **Occurrence** | - | *0.00** | 0.11 | *0.00** | 0.87 | *0.00** | *0.01** | *0.00** | 0.71 | 0.48 |
| **B** | **Total**  **Rainfall** | 0.44 | - | *0.04** | *0.00** | 0.11 | *0.00** | 0.15 | *0.00** | 0.56 | 0.60 |
| **C** | **Maximum Temperature** | 0.10 | -0.12 | - | *0.00** | *0.00** | *0.00** | 0.66 | 0.53 | *0.01** | 0.64 |
| **D** | **Minimum Temperature** | 0.29 | 0.51 | 0.39 | - | *0.00** | *0.00** | *0.01** | *0.00** | *0.00** | 0.66 |
| **E** | **Average**  **Temperature** | 0.10 | 0.09 | 0.83 | 0.72 | - | *0.00** | 0.10 | *0.00** | *0.00** | 0.93 |
| **F** | **Relative**  **Humidity** | 0.37 | 0.78 | -0.43 | 0.36 | -0.18 | - | 0.55 | *0.00** | 0.39 | 0.65 |
| **G** | **Average Wind**  **Speed** | 0.17 | 0.08 | 0.02 | 0.14 | 0.09 | -0.03 | - | *0.00** | *0.00** | 0.34 |
| **H** | **Minimum Wind Direction** | 0.27 | 0.35 | 0.04 | 0.41 | 0.25 | 0.33 | 0.24 | - | *0.00** | 0.06 |
| **I** | **Maximum Wind Direction** | -0.23 | -0.03 | -0.16 | -0.24 | -0.25 | 0.05 | -0.17 | -0.19 | - | *0.04** |
| **J** | **Southern Oscillation Index** | -0.05 | -0.03 | 0.03 | 0.03 | 0.01 | 0.03 | -0.05 | -0.11 | 0.12 | - |

*Lower triangle represents the correlation coefficient while Upper triangle represents the p-value.*

** statistically significant variables*

*Underlined variable is observed to be highly correlated*

**Table S1b. Correlation Analysis among Lagged Meteorological factors (LG)**

| **Meteorological Factors** | | **A** | **B** | **C** | **D** | **E** | **F** | **G** | **H** | **I** |
| --- | --- | --- | --- | --- | --- | --- | --- | --- | --- | --- |
| **A** | **Flood**  **Occurrence** | - | *0.00** | *0.00** | *0.00** | *0.00** | *0.00** | *0.00** | 0.17 | 0.95 |
| **B** | **Total**  **Rainfall** | 0.26 | - | *0.00** | *0.00** | *0.00** | *0.00** | *0.00** | 0.93 | 0.39 |
| **C** | **Maximum Temperature** | 0.20 | 0.33 | - | *0.00** | *0.00** | *0.00** | *0.00** | 0.17 | 0.41 |
| **D** | **Minimum Temperature** | 0.32 | 0.30 | 0.51 | - | *0.00** | *0.00** | *0.00** | 0.21 | 0.85 |
| **E** | **Average**  **Temperature** | 0.26 | 0.32 | 0.69 | 0.63 | - | *0.00** | *0.00** | 0.08 | 0.85 |
| **F** | **Relative**  **Humidity** | 0.35 | 0.39 | 0.47 | 0.65 | 0.58 | - | *0.00** | 0.48 | 0.08 |
| **G** | **Minimum Wind Direction** | 0.36 | 0.22 | 0.22 | 0.37 | 0.27 | 0.40 | - | 0.17 | 0.08 |
| **H** | **Maximum Wind Direction** | 0.09 | -0.01 | -0.09 | -0.08 | -0.12 | -0.05 | -0.09 | - | 0.36 |
| **I** | **Southern Oscillation Index** | -0.01 | -0.60 | -0.05 | 0.01 | -0.63 | 0.12 | -0.11 | 0.06 | - |

*Lower triangle represents the correlation coefficient while Upper triangle represents the p-value.*

** statistically significant variables*
