# Supplementary material for: Machine learning methods reveal the temporal pattern of dengue incidence using meteorological factors in metropolitan Manila, Philippines
Source: BMC Infect Dis. 2018 Apr 17;18:183. doi: 10.1186/s12879-018-3066-0 (PMC5905126; doi:10.1186/s12879-018-3066-0)
Supplement: Supplementary file 2 — Table S2a. Adjusted R-square and Akaike Information Criterion of Meteorological Factors (MF) using Generalized Additive Modeling (GAM). Table S2b. Adjusted R-square and Akaike Information Criterion of Lagged Meteorological Factors (LG) using Generalized Additive Modeling (GAM). Table S2c. Model Quality of Seasonal Autoregressive Integrated Moving Average (SARIMA) Model ((p,d,q)(P,D,Q)m) with Meteorological Factors (MF) as Exogenous Variables based on the Akaike Information Criterion. Table S2d. Model Quality of Seasonal Autoregressive Integrated Moving Average (SARIMA) Model ((p,d,q)(P,D,Q)m) with Lagged Meteorological Factors (LG) as Exogenous Variables based on the Akaike Information Criterion (DOCX 21 kb) [file 12879_2018_3066_MOESM2_ESM.docx]

**Table S2a. Adjusted R-square and Akaike Information Criterion of Meteorological Factors (MF) using Generalized Additive Modeling (GAM)**

| **Meteorological Factors (MF)** | **R-Square (adj)** | **AIC** |
| --- | --- | --- |
| All Meteorological Factors | 0.45 | 286.18 |
| Minimum+Average+Maximum Temperatures+ Southern Oscillation Index | 0.39 | 297.34 |
| Relative Humidity | 0.27 | 324.27 |
| Flood+Total Rainfall+Relative Humidity | 0.28 | 325.41 |
| Maximum Temperature | 0.26 | 328.69 |
| Mean Temperature | 0.21 | 344.69 |
| Rainfall | 0.16 | 355.17 |
| Minimum Temperature | 0.13 | 364.66 |
| Average Wind Speed+Minimum+Maximum Wind Directions | 0.14 | 367.97 |
| Flood | 0.08 | 373.08 |
| Minimum Wind Speed | 0.08 | 373.37 |
| Wind Speed | 0.04 | 386.05 |
| Southern Oscillation Index | 0.02 | 386.38 |
| Maximum Wind Speed | 0.01 | 389.26 |

**Table S2b. Adjusted R-square and Akaike Information Criterion of Lagged Meteorological Factors (LG) using Generalized Additive Modeling (GAM)**

| **Lagged Meteorological Factors (LG)** | **R-Square (adj)** | **AIC** |
| --- | --- | --- |
| All Meteorological Factors | 0.67 | 145.81 |
| Minimum+Average+Maximum Temperatures+ Southern Oscillation Index | 0.52 | 203.63 |
| Flood+Total Rainfall+Relative Humidity | 0.52 | 238.42 |
| Mean Temperature | 0.38 | 255.96 |
| Maximum Temperature | 0.34 | 265.89 |
| Relative Humidity | 0.43 | 268.71 |
| Minimum Temperature | 0.36 | 274.83 |
| Rainfall | 0.38 | 290.87 |
| Average Wind Speed+Minimum+Maximum Wind Directions | 0.13 | 311.52 |
| Maximum Wind Speed | 0.03 | 330.09 |
| Southern Oscillation Index | 0.03 | 332.93 |
| Flood | 0.14 | 350.13 |
| Minimum Wind Speed | 0.1 | 350.92 |

**Table S2c. Model Quality of Seasonal Autoregressive Integrated Moving Average (SARIMA) Model ((p,d,q)(P,D,Q)m) with Meteorological Factors (MF) as Exogenous Variables based on the Akaike Information Criterion**

| **Model with Exogenous Variables (MF factors)** | | **Best Model** | **AIC** |
| --- | --- | --- | --- |
| Dengue Incidence | - | (0,1,0)(0,0,1)[52] | -144.72 |
| Dengue Incidence | All Meteorological Factors | (2,1,3)(1,0,0)[52] | -200.67 |
| Dengue Incidence | Average Wind Speed+Minimum+Maximum Wind Directions | (2,1,2)(1,0,0)[52] | -201.75 |
| Dengue Incidence | Total Rainfall | (3,1,2)(1,0,0)[52] | -202.86 |
| Dengue Incidence | Flood | (3,1,2)(1,0,0)[52] | -202.99 |
| Dengue Incidence | Minimum Wind Direction | (3,1,2)(1,0,0)[52] | -204.39 |
| Dengue Incidence | Southern Oscillation Index | (0,1,0)(1,0,0)[52] | -204.54 |
| Dengue Incidence | Minimum+Average+Maximum Temperatures+ Southern Oscillation Index | (3,1,3)(1,0,0)[52] | -204.63 |
| Dengue Incidence | Average Temperature | (0,1,0)(0,0,1)[52] | -204.65 |
| Dengue Incidence | Average Wind Speed | (3,1,2)(1,0,0)[52] | -205.23 |
| Dengue Incidence | Maximum Temperature | (3,1,2)(1,0,0)[52] | -205.30 |
| Dengue Incidence | Maximum Wind Direction | (0,1,0)(0,0,1)[52] | -206.27 |
| Dengue Incidence | Minimum Temperature | (3,1,0)(1,0,0)[52] | -207.17 |
| Dengue Incidence | Flood+Total Rainfall+Relative Humidity | (3,1,2)(1,0,0)[52] | -207.31 |
| Dengue Incidence | Relative Humidity | (0,1,0)(0,0,1)[52] | -209.22 |

**Table S2d. Model Quality of Seasonal Autoregressive Integrated Moving Average (SARIMA) Model ((p,d,q)(P,D,Q)m) with Lagged Meteorological Factors (LG) as Exogenous Variables based on the Akaike Information Criterion**

| **Model with Exogenous Variables (LG factors)** | | **Best Model** | **AIC** |
| --- | --- | --- | --- |
| Dengue Incidence | All Meteorological Factors | (0,1,0)(1,0,0)[52] | -125.94 |
| Dengue Incidence | Minimum+Maximum Wind Directions | (4,1,0)(1,0,0)[52] | -191.80 |
| Dengue Incidence | Maximum Wind Direction | (4,1,0)(1,0,0)[52] | -193.34 |
| Dengue Incidence | Maximum Temperature | (0,1,0)(1,0,0)[52] | -200.35 |
| Dengue Incidence | Minimum Temperature | (0,1,0)(1,0,0)[52] | -201.04 |
| Dengue Incidence | Southern Oscillation Index | (3,1,0)(1,0,0)[52] | -201.61 |
| Dengue Incidence | Minimum+Average+Maximum Temperatures+ Southern Oscillation Index | (0,1,0)(0,0,1)[52] | -203.27 |
| Dengue Incidence | Minimum Wind Direction | (0,1,0)(1,0,0)[52] | -204.54 |
| Dengue Incidence | Flood | (0,1,0)(1,0,0)[52] | -208.06 |
| Dengue Incidence | Flood+Total Rainfall+Relative Humidity | (0,1,0)(1,0,0)[52] | -208.35 |
| Dengue Incidence | Total Rainfall | (0,1,0)(1,0,0)[52] | -209.56 |
| Dengue Incidence | Relative Humidity | (0,1,0)(1,0,0)[52] | -212.47 |
| Dengue Incidence | Average Temperature | (0,1,0)(1,0,0)[52] | -212.82 |
