# Supplementary material for: Machine learning methods reveal the temporal pattern of dengue incidence using meteorological factors in metropolitan Manila, Philippines
Source: BMC Infect Dis. 2018 Apr 17;18:183. doi: 10.1186/s12879-018-3066-0 (PMC5905126; doi:10.1186/s12879-018-3066-0)
Supplement: Supplementary file 3 — Table S3a. Statistical significant Meteorological (MF) and its Lagged (LG) Factors in General Additive Modeling (GAM) and Seasonal Autoregressive Integrated Moving Average with Exogenous Variables (SARIMAX). Table S3b. Variable Importance of Random Forest and Gradient Boosting in Meteorological factors (MF) and its corresponding lags (LG). (DOCX 21 kb) [file 12879_2018_3066_MOESM3_ESM.docx]

**Table S3a. Statistical significant Meteorological (MF) and its Lagged (LG) Factors in General Additive Modeling (GAM) and Seasonal Autoregressive Integrated Moving Average with Exogenous Variables (SARIMAX)**

| **Meteorological Variables** | **GAM** | | | | **SARIMA** | | | |
| --- | --- | --- | --- | --- | --- | --- | --- | --- |
|  | **MF** | | **LG** | | **MF** | | **LG** | |
|  | F-value | p-value | F-value | p-  value | z-value | p-value | z-value | p-value |
| Flood  Occurrence | 1.737 | 0.08 | 2.80 | *0.01** | -0.60 | 0.55 | 1.91 | *0.05** |
| Total  Rainfall | 0.846 | 0.45 | 4.77 | *0.01** | 0.75 | 0.46 | 1.41 | 0.16 |
| Maximum  Temperature | 4.459 | *0.00** | 3.93 | *0.02** | 1.74 | 0.08 | 0.84 | 0.40 |
| Minimum  Temperature | 0.886 | 0.44 | 2.00 | 0.16 | -2.62 | *0.01** | 0.63 | 0.53 |
| Average  Temperature | 1.597 | 0.19 | 1.98 | 0.10 | 0.81 | 0.42 | 46.64 | *0.00** |
| Relative  Humidity | 4.091 | *0.04** | 11.28 | *0.00** | -0.76 | 0.44 | 0.39 | 0.70 |
| Average Wind  Speed | 1.605 | 0.15 | - | - | 2.49 | *0.01** | - | - |
| Minimum Wind  Direction | 3.144 | 0.09 | 1.59 | 0.13 | 1.36 | 0.17 | 1.01 | 0.31 |
| Maximum Wind Direction | 1.165 | 0.28 | 1.64 | 0.18 | -3.03 | *0.00** | - | - |
| Southern Oscillation Index | 7.851 | *0.01** | 20.04 | *0.00** | 0.16 | 0.87 | 1.35 | 0.18 |

** statistically significant variables*

**Table S3b. Variable Importance of Random Forest and Gradient Boosting in Meteorological factors (MF) and its corresponding lags (LG).**

| **METEOROLOGICAL VARIABLES** | **METEOROLOGICAL**  **FACTORS (MF)** | | | **LAGGED METEOROLOGICAL FACTORS (LG)** | | |
| --- | --- | --- | --- | --- | --- | --- |
|  | RF | | GB | RF | | GB |
|  | %Inc  MSE | Inc  Node | Relative Contribution (%) | %Inc  MSE | Inc  Node | Relative Contribution (%) |
| Flood  Occurrence | 9.56 | 1.56 | 1.73 | 10.01 | 1.79 | 1.88 |
| Total  Rainfall | ***15.16*** | 9.24 | ***11.63*** | ***26.86*** | 10.46 | 10.49 |
| Maximum Temperature | ***27.70*** | 13.94 | ***28.32*** | ***22.05*** | 12.55 | ***22.32*** |
| Average Temperature | ***16.41*** | 10.67 | ***11.61*** | ***23.12*** | 11.66 | ***19.24*** |
| Minimum Temperature | ***14.81*** | 7.11 | 6.85 | ***24.52*** | 9.80 | 8.78 |
| Relative  Humidity | ***18.45*** | 13.71 | ***20.45*** | ***32.47*** | 17.27 | ***14.36*** |
| Wind  Speed | 7.01 | 2.99 | 3.47 | - | - | - |
| Minimum Wind Direction | 6.24 | 4.51 | 4.85 | 10.53 | 3.11 | 3.60 |
| Maximum Wind Direction | 1.92 | 2.66 | 1.91 | 8.25 | 2.12 | 6.76 |
| Southern Oscillation Index | 6.36 | 6.30 | 9.18 | ***17.97*** | 5.65 | ***12.57*** |

*Note: Variable values that are bold, italicized and underlined are deemed to be important based on the computed randomness threshold for MF (>10%) and LG (>11%) datasets.*
