# Supplementary material for: Machine learning methods reveal the temporal pattern of dengue incidence using meteorological factors in metropolitan Manila, Philippines
Source: BMC Infect Dis. 2018 Apr 17;18:183. doi: 10.1186/s12879-018-3066-0 (PMC5905126; doi:10.1186/s12879-018-3066-0)
Supplement: Supplementary file 4 — Table S4a. Comparison of Model Quality and Performance in Meteorological Factors (MF) dataset when Maximum, Average Temperatures and Relative Humidity are excluded in the Model Development. Table S4b. Comparison of Model Quality and Performance in Lagged Meteorological Factors (LG) dataset when Maximum, Average Temperatures and Relative Humidity are excluded in the Model Development. (DOCX 13 kb) [file 12879_2018_3066_MOESM4_ESM.docx]

**SUPPLEMENTARY MATERIAL TABLE 4**

**Supplemental Table 4a. Comparison of Model Quality and Performance in Meteorological Factors (MF) dataset when Maximum, Average Temperatures and Relative Humidity are excluded in the Model Development**

| **Modeling Approaches** | | All Meteorological Variables | Exclude Maximum Temperature | Exclude Average Temperature | Exclude Relative Humidity |
| --- | --- | --- | --- | --- | --- |
| GAM | AIC | 286.18 | 300.01 | 288.57 | 287.02 |
|  | RMSE | 0.33 | 0.33 | 0.37 | 0.34 |
|  | MAE | 0.27 | 0.27 | 0.27 | 0.28 |
| SARIMAX | AIC | -200.67 | -202.9 | -206.74 | -201.71 |
|  | RMSE | 0.42 | 0.46 | 0.67 | 0.60 |
|  | MAE | 0.39 | 0.42 | 0.54 | 0.48 |
| RF | RMSE | 0.29 | 0.30 | 0.31 | 0.29 |
|  | MAE | 0.23 | 0.24 | 0.25 | 0.23 |
| GB | RMSE | 0.30 | 0.30 | 0.31 | 0.31 |
|  | MAE | 0.24 | 0.25 | 0.25 | 0.25 |

**Supplemental Table 4b. Comparison of Model Quality and Performance in Lagged Meteorological Factors (LG) dataset when Maximum, Average Temperatures and Relative Humidity are excluded in the Model Development**

| **Modeling Approaches** | | All Lagged Meteorological Variables | Exclude Maximum Temperature | Exclude Average Temperature | Exclude Relative Humidity |
| --- | --- | --- | --- | --- | --- |
| GAM | AIC | 145.81 | 154.00 | 147.91 | 169.39 |
|  | RMSE | 0.22 | 0.23 | 0.23 | 0.24 |
|  | MAE | 0.17 | 0.17 | 0.17 | 0.18 |
| SARIMAX | AIC | -125.94 | -127.89 | -128.66 | -127.86 |
|  | RMSE | 0.31 | 0.31 | 0.35 | 0.31 |
|  | MAE | 0.27 | 0.27 | 0.30 | 0.28 |
| RF | RMSE | 0.21 | 0.21 | 0.21 | 0.24 |
|  | MAE | 0.15 | 0.15 | 0.15 | 0.17 |
| GB | RMSE | 0.23 | 0.23 | 0.23 | 0.28 |
|  | MAE | 0.17 | 0.18 | 0.17 | 0.21 |
